# Association of *FBN1* polymorphism with susceptibility of adolescent idiopathic scoliosis: a case-control study

**DOI:** 10.1186/s12891-022-05370-1

**Published:** 2022-05-07

**Authors:** Gustavo Borges Laurindo de Azevedo, Jamila Alessandra Perini, Antônio Eulálio Pedrosa Araújo Junior, Luis Antonio Medeiros Moliterno, Rodrigo Mantelatto Andrande, João Antonio Matheus Guimarães, Helton Luiz Aparecido Defino

**Affiliations:** 1 Spine Surgery Center, National Institute of Traumatology and Orthopaedics (INTO), Rio de Janeiro, RJ Brazil; 2 Departments of Orthopaedic and Anesthesiology, Ribeirão Preto Medical School, University of São Paulo, de São Paulo, Brazil; 3 Research Division, National Institute of Traumatology and Orthopaedics (INTO), Avenida Brasil, 500, Rio de Janeiro, RJ zip code 20940-070 Brazil; 4 Research Laboratory of Pharmaceutical Sciences (LAPESF), State University of Rio de Janeiro (UERJ), Rio de Janeiro, RJ Brazil

**Keywords:** Adolescent idiopathic scoliosis, Fibrillin-1, Polymorphism

## Abstract

**Background:**

Fibrillin-1 (FBN1) is an extracellular matrix glycoprotein essential to the structural component of microfibrils and *FBN1* gene polymorphisms can be associated with adolescent idiopathic scoliosis (AIS) susceptibility. This study aimed to evaluate the potential role of the *FBN1* rs12916536 polymorphism in AIS development or severity and the variation in Cobb angle in relation to patient’s characteristics.

**Methods:**

DNA from 563 subjects (185 AIS patients and 378 controls) were genotyped using a validated TaqMan allelic discrimination assay. A multivariate logistic regression model evaluated the association between polymorphism and AIS, using the adjusted odds ratios (OR) with their respective 95% confidence intervals (95% CI). A linear regression analysis evaluated the variation in Cobb angle according to the patient’s age and body mass index (BMI).

**Results:**

Among the AIS group there was a predominance of females (12:1), low or normal BMI (90%), 58% had a Cobb angle greater than 45° and 74% were skeletally mature. Age was a risk factor (4-fold) for curve progression higher than BMI (*P* < 0.001). The allelic frequency of the rs12916536 *G > A* polymorphism was 40% in controls and 31% in AIS cases; and this difference was statistically significant (*P* = 0.004). *FBN1* rs12916536 *GA + AA* genotypes were associated with a lower risk of AIS susceptibility (OR = 0.58 and 95% CI = 0.35–0.98), after adjustment for age, sex and BMI. However, no significant differences were detected in polymorphism distribution with the severity of the disease (Cobb < 45° or ≥ 45°).

**Conclusion:**

Age was a risk factor for progression of the scoliotic curve and *FBN1* rs12916536 polymorphism a protective factor for AIS susceptibility.

**Supplementary Information:**

The online version contains supplementary material available at 10.1186/s12891-022-05370-1.

## Introduction

Adolescent idiopathic scoliosis (AIS), a three-dimensional deformity of the spine, typically becomes evident near the onset of puberty, with no apparent cause and prevalence around 3% [1]. Most patients have non-progressive deformity, and factors associated with curve progression and disease development are not fully understood. Thus, several distinct pathogenesis theories have been proposed for AIS development, including biomechanical, neurological, hormonal, growth-related and genetic [2–5].

Genome-wide association studies (GWAS) have identified variants in genes related to muscle, cartilage, bone, intervertebral discs, and connective tissue development that appear to be associated with AIS susceptibility [6–17]. However, the molecular genetic origin of AIS development remains unknown. GWAS of rare variants using exome sequence analysis identified fibrillin-1 (FBN1) as a susceptible gene of AIS [9]. In addition, common variant rs12916536 of *FBN1* was significantly associated with AIS development in Chinese population, suggested a regulatory role in the expression of gene [18].

The *FBN1* gene, localized at chromosome 15q21.1, comprising aroud 200 kb and containing 65 exons [19], codes for an extracellular matrix glycoprotein crucial to extracellular microfibrils organization in skeletal muscle cells and dermal fibroblasts. Spinal stability needs the biomechanical properties of the ligaments, discs and connective-tissue components [20, 21]. Several inherited connective tissue disorders (e.g., syndromes: Marfan, Ehlers-Danlos, Beals and Weill-Marchesani and osteogenesis imperfecta) that show clinical features of structural scoliosis were associated with rare mutations in *FBN1* [20–25]. As far as we know, no studies have investigated the association between common genetic variants of *FBN1* and AIS in Brazilians. This population is extraordinarily heterogenic and extrapolation of genetic data from well-defined ethnic groups may not be applicable to most Brazilians [26–28].

Therefore, due to the relationship between genetic variants in connective-tissue genes and scoliosis, this study was designed to investigate the association between the *FBN1* rs12916536 polymorphism and the susceptibility of AIS in Brazilians, as well as its influence on the severity of the disease. In addition, was evaluate the variation in Cobb angle in dependence to the patient’s age and BMI.

## Materials and methods

### Study population

The current study comprises a retrospective case-control evaluation of 563 subjects (185 AIS patients and 378 controls) from a reference center in Orthopedics. The study protocol was approved by the Human Research Ethics Committees of the *Instituto Nacional de Traumatologia e Ortopedia Jamil Haddad* – INTO (protocol numbers 637.973 and 2.767.503), and all subjects provided written informed consent. The study was conducted in accordance with the Helsinki Declaration. All sociodemographic and clinical data were obtained during the recruitment process between 2018 and 2020. The body mass index (BMI) was calculated as the weight status (kg) divided by the square of height (m^2^) and the weight status is classified into five groups: underweight (BMI < 18.5), normal weight (18.5 ≤ BMI ≤ 24.9), overweight (25 ≤ BMI ≤ 29.9), obesity (30 ≤ BMI < 40) and morbid obesity (BMI ≥ 40).

All patients had idiopathic scoliosis diagnosed by clinical and radiographic examination with the spinal curve Cobb angle measuring ≥10°. Posteroanterior radiographs were used to measure the major curve angles employing the Cobb method [29]. AIS cases were separated into two groups: Cobb < 45° and Cobb ≥45° according to the magnitude of the Cobb angle and indication of surgical treatment for scoliosis. The Risser sign was used to estimate skeletal maturity and is a predictor of scoliosis progression [30]. The skeletal maturity was assessed by evaluating ossification of the iliac apophysis and patients with a Risser’s sign of zero to 3 were classified as skeletally immature and those with a Risser’s sign of 4 or 5 were classified as skeletally mature. All measurements were independently made by two investigators (GBLA and AEPAJ or LAMM) who are experienced spine surgeons and blinded to the clinical information to avoid bias.

The control group (*N* = 378) consisted of healthy volunteers recruited at INTO’s blood bank when they appeared to donate blood. An orthopedic spine surgeon evaluated them to rule out any spine deformity.

### Polymorphisms genotyping

Genomic DNA was obtained from oral mucosa collected by swab or from peripheral blood samples using an extraction kit (Qiagen) following the procedures recommended by the manufacturer. The genotyping analyses of *FBN1* rs12916536 polymorphism (chr15:48414374) were performed using a *TaqMan* allelic discrimination assay (C__31343379_20) by 7500 Real-Time System (Applied Biosystems, Foster City, CA, USA). PCR amplification was performed in 8 μL reactions with 30 ng of template DNA, 1x TaqMan Universal Master Mix, 1x each primer and probe assay. Thermal cycling was initiated with a first denaturation step of 10 min at 95 °C, followed by 40 cycles of denaturation at 92 °C for 15 s and annealing at 60 °C for 1 min. To assure genotyping quality, in each reaction, two standardized negative and positive controls of each polymorphism genotype were used, as previously described [28].

### Statistical analysis

A sample size calculation was performed using Epi Info 7, version 7.2.4.0 (http://wwwn.cdc.gov/epiinfo/html/downloads.htm) capable of detecting differences between cases and controls, assuming an odds ratio of 0.5 with a power of 0.8 and 5% type I error.

A descriptive study of the population was conducted, presenting relative frequencies for each categorical variable. The categorical data were expressed as percentages and evaluated by the Chi-square (χ2) test or Fisher’s exact test, when applicable. A linear regression analysis was performed using Cobb angle and age or BMI to evaluate the variation in Cobb angle in relation to the patient’s age and BMI.

Genotypic frequency of *FBN1* polymorphism was derived by direct gene counting, and the adherence to the Hardy–Weinberg principle was evaluated by the Chi-square test for goodness-of-fit. The magnitude of association from each comparison was estimated by calculating crude odds ratios (ORs) with 95% confidence intervals (95% CIs). As a final regression model used to control possible confounding factors (sociodemographic and clinical features), each variable was introduced considering the biological and statistical significance of the univariate analysis, which an input significance level less than 0.25 (*P* ≤ 0.25) and output significance was 0.05 (*P* ≤ 0.05) at the regression model, as previously described [28, 31]. The difference was statistically significant when *P* < 0.05. All analyses were performed using the Statistical Package for Social Sciences (SPSS Inc., Chicago, IL, USA, version 20.0).

## Results

Table 1 presents the main sociodemographic and clinical characteristics of the AIS case population. The mean age and BMI (and ranges) were 18.6 ± 6.7 (10–48) years and 20.4 ± 4.0 (14.0–39.9) kg/m^2^, respectively. There was a predominance (89.6%) of low or normal BMI values (≤24.9) among the AIS patients group and the ratio of female to male patients affected by the disease was 12.2:1. Of the 185 AIS patients, 129 (73.7%) were classified as skeletally mature, according to the Risser grade. The mean major spinal curve magnitude was 47.7 ± 16.3 (11–110) degrees and 58% (*N* = 102) had curves greater than 45°.

**Table 1:** Demographics and clinical characteristics of 185 patients with adolescent idiopathic scoliosis

Variables	Idiopathic scoliosis cases
	N (%)
**Age**^a,b^ (years old)	
≤ 18	115 (62.8)
19 a 28	51 (27.9)
≥ 29	17 (9.3)
**Sex**	
Female	171 (92.4)
Male	14 (7.6)
**BMI**^c^ (kg/m^2^)	
≤ 18.50	64 (35.2)
18.51 a 24.9	99 (54.4)
25.0 a 29.9	12 (6.6)
≥ 30	7 (3.8)
**Risser scale**^d^	
Immature (0-III)	46 (26.3)
Mature (IV-V)	129 (73.7)
**Cobb (degrees)**^e^	
< 45	74 (42.0)
**≥** 45	102 (58.0)

^a^Information obtained from 183 cases

^b^Values categorized according to the quartile distribution of the total study population (*N* = 563)

^c^Information obtained from 182 cases

^d^Information obtained from 175 cases

^e^Information obtained from 176 cases

The mean age in AIS patients with Cobb < 45° (15.6 ± 6.3 years 10–29) was significantly smaller (*P* < 0.001) than patients with Cobb ≥45° (22.5 ± 6.7 years 11–48).

**Fig. 1:**
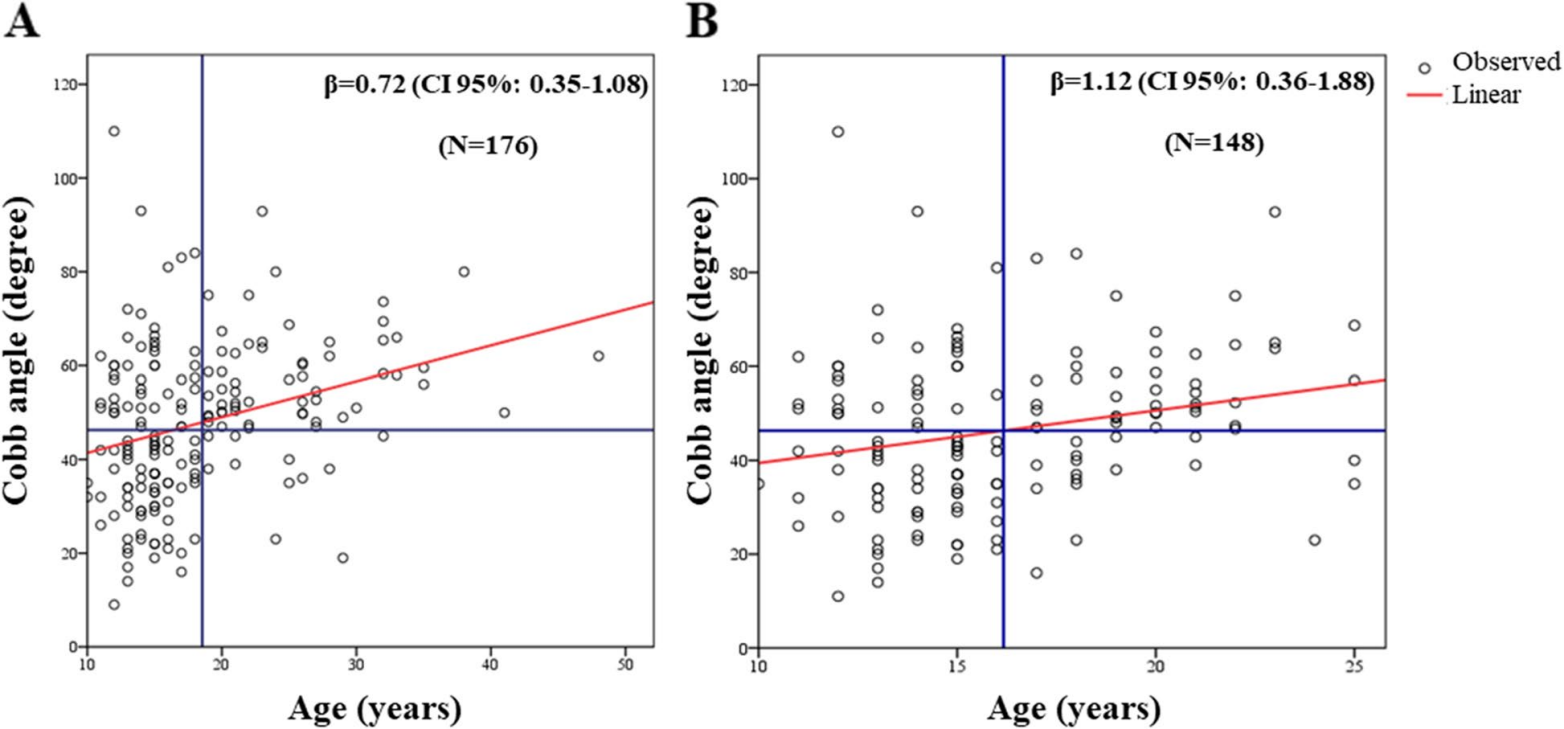
Relationship between the Cobb angle and the age in adolescent idiopathic scoliosis. Legend: The circles represent each patients. The red line is linear function coefficient (**A**, R^2^ = 0.09 with *P* < 0.001 and **B**, R^2^ = 0.05 with *P* = 0.004) and the blue lines are means of th age and Cobb angle. Information obtained from 176 cases (**A**) and 148 AIS patients up to 25 years of age (**B**)

The statistical analysis estimated a mean increase in the Cobb angle of 0.72° and 1.12° for every additional year of age, considering all AIS cases and only patients up to 25 years of age, respectively. The relative regression lines, with 95% confidence intervals for the estimated mean Cobb angle values, are shown in Fig. 1A and B. The comparison of the standardized regression coefficients β, which determines the contribution linked to various independent variables expressed in different measurement scales, showed that age contributed 4-fold more than BMI to the Cobb angle increase (Table 2).

**Table 2:** Linear regression analysis of the Cobb angle with age and BMI of adolescent idiopathic scoliosis patients

Variables	Regression Coefficient	***P***-value	Standardized coefficient β	(CI 95%)
**Cobb (*N*** **= 176)**				
**Age**	0.72	**< 0.001**	0.29	0.35–1.08
**Sex**	−6.21	0.17	−0.10	−15.12 – 2.71
**BMI**	0.33	0.31	0.08	−0.30 – 0.96
**r**^**2**^ **ajusted**		0.11		
***P*-model**^**a**^		**< 0.001**		

^a^*P*-model calculated by ANOVA test. Information obtained from 176 cases

**Fig. 2:**
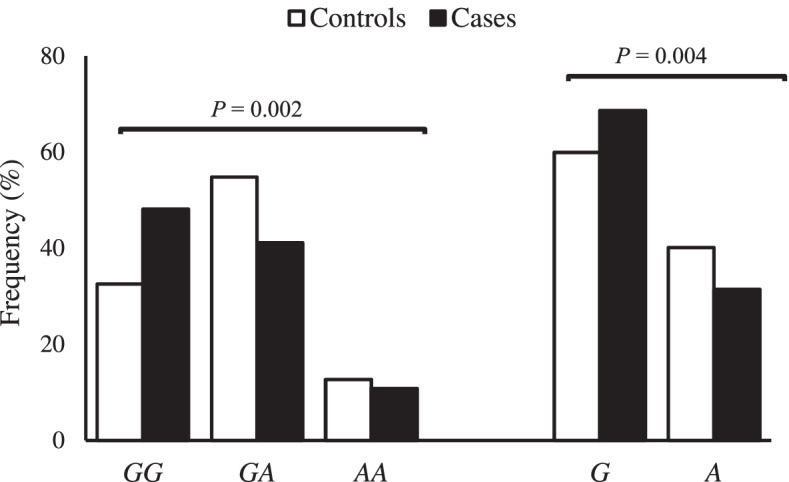
Allelic and genotypic distribution of the *FBN1 rs12916536* polymorphism in adolescent idiopathic scoliosis cases and controls. Legend: Number of controls = 378 and cases = 185. *P*-value calculated by Chi-Square Teste or Fisher’s exact test, when necessary

The rate of successful genotyping was 100% and the genotypic distribution in the entire study population was in Hardy-Weinberg equilibrium. A statistically different frequency was observed between AIS cases and controls in genotype and allele comparison of the *FBN1* rs12916536 *G > A* polymorphism (Fig. 2). After adjustment for confounding factors (age, sex and BMI), the *FBN1* rs12916536 genotypes (GA + AA) were associated with a lower risk of developing AIS, since the variant allele was more frequent in the control group. No significant associations were found in *FBN1* rs12916536 polymorphism with severity of the disease, considering either AIS cases with a Cobb angle < 45° or a Cobb angle ≥45° (Table 3).

**Table 3:** Association between the *FBN1* rs12916536 polymorphism with adolescent idiopathic scoliosis and its severity (*N* = 563)

***FBN1***	Controls	All cases	OR ajusted^**b**^	Cobb < 45°	OR ajusted^**c**^	Cobb ≥ 45°	OR ajusted^**d**^	OR ajusted^**e**^
rs12916536	(***N*** = 378)	(***N*** = 185)	(CI 95%)	(***N*** = 74)	(CI 95%)	(***N*** = 102)	(CI 95%)	(CI 95%)
** N (%) N (%) N (%)**								
*GG*	123 (32.5)	89 (48.1)	1^a^	37 (50.0)	1^a^	48 (47.1)	1^a^	1^a^
*GA*	207 (54.8)	76 (41.1)	**0.52 (0.30–0.90)**	29 (39.2)	0.54 (0.22–1.33)	43 (42.1)	0.68 (0.37–1.24)	1.12 (0.55–2.26)
*AA*	48 (12.7)	20 (10.8)	0.95 (0.40–2.26)	8 (10.8)	2.40 (0.45–12.75)	11 (10.8)	0.91 (0.34–2.45)	1.28 (0.42–3.90)
*GG*	123 (32.5)	89 (48.1)	1^a^	37 (50.0)	1^a^	48 (47.1)	1^a^	1^a^
*GA + AA*	255 (67.5)	96 (51.9)	**0.58 (0.35–0.98)**	37 (50.0)	0.65 (0.28–1.55)	54 (52.9)	0.71 (0.40–1.27)	1.15 (0.60–2.23)
*G*	453 (59.9)	254 (68.6)	1^a^	103 (69.6)	1^a^	139 (68.1)	1^a^	1^a^
*A*	303 (40.1)	116 (31.4)	0.79 (0.54–1.16)	45 (30.4)	0.96 (0.51–1.81)	65 (31.9)	0.85 (0.56–1.30)	1.14 (0.69–1.88)

*OR* Odds ratio, ajusted by age, sex and BMI, *CI* confidence interval 95%

^a^Reference group

^b^Association analysis between controls and all AIS cases

^c^Association analysis between controls and Cobb < 45° AIS cases

^d^Association analysis between controls and Cobb ≥45° AIS cases

^e^Association analysis between Cobb < 45° and Cobb ≥45° AIS cases

## Discussion

The *FBN1* rs12916536 polymorphism was associated with the susceptibility of AIS in the Brazilian population, but it was not influence on the severity of the disease. Age was an independent risk factor for scoliotic curve progression. The molecular mechanisms involved in the pathogenesis and development of AIS have been the subject of many research studies; however, the etiology remains an enigma [5, 32, 33]. For example, low BMI has been associated with AIS and it is hypothesized that this association may be related to the pathogenesis of the disease [34–38]. The majority of our patients (~ 90%) had low or normal BMI and 58% had curves with Cobb angles exceeding 45°, a parameter commonly used to indicate the need for surgical correction of scoliosis [39]. Our results corroborate with Miyagi and colleagues, as patients with curves with Cobb angles exceeding 45° had significantly lower BMI than patients with curves under 45° [35]. Furthermore, of the significant correlations between anthropometric parameters and the scoliotic curve severity [34], low BMI also was associated with increased risk of all poor outcomes, including brace treatment failure and the need for surgical intervention [37].

The risk of scoliosis curve progression was also associated with female sex, age, skeletal maturity and curve Cobb angle exceeding 30° at presentation [39–41]. The female-to-male ratio (12:1) observed here is consistent with previous studies [9, 42]. In addition, most of our AIS patients were skeletally mature with curve Cobb angles exceeding 45°. This is likely due to the fact that all cases were recruited at a public orthopedic referral hospital in Brazil, where the majority of patients were referred from other facilities for a surgical indication and sometimes the waiting times were surgery were long. Thus, age contributed 4-fold more than BMI to the Cobb angle increase in our cases. Our results corroborate with previous findings that show progression of curves above 30° in skeletally mature patients at rates of < 1° per year [39, 43, 44]. However, patients with Cobb angles exceeding 45° were more likely come back for follow up and the association found might just be due to follow-up bias. So, intervening early is vital because patients with curves below 30° can have a quality of life similar to a healthy population [44, 45] and they are amenable for brace treatment, which was not the case for most of our patients.

Genetic factors have been largely considered to play an important role in its onset and progression of AIS [5]. Rare variants in *FBN1* were first identified in severe AIS North American patients with European ancestry (*n* = 344), other ancestral backgrounds (*n* = 47) and replicated in an independent cohort of Han Chinese (*n* = 370), suggesting that this variant can be predictive of curve severity and promising as a new option for early diagnosis and more timely treatment of severe AIS [9]. *FBN1* variants were also associated with Marfan syndrome’s skeletal features, autosomal dominant disorder, which frequently requires orthopedic surgical intervention [25, 46]. FBN1 is richly distributed in structural elements of elastic and non-elastic tissues, responsible for connective tissue disorders, such as scoliosis [46, 47]. Furthermore, *FBN1* variants can influence in upregulated transforming growth factor-beta signaling in paravertebral muscles [48, 49].

AIS patients showed significantly decreased *FBN1* mRNA expression and common variants (polymorphisms) of *FBN1* were associated with increased individual risk of AIS [18]. The inheritable susceptibility to AIS justifies the growing interest in identifying genetic polymorphisms that could lead to an increased risk or severity of the disease [5]. The present results indicate a negative association between *FBN1* rs12916536 *G > A* and the risk of developing AIS, since cases showed a significantly lower frequency of genotypes *GA + AA* than the controls. This polymorphism probably plays an important role in the regulation of *FBN1* expression, since it was marked by enhancer histone in multiple cell lines [50]. It is noteworthy that our result is in agreement with Sheng and colleagues [18], who found that *FBN1* rs12916536 was associated with the development of AIS in a large study (952 cases and 1499 controls) of Chinese.

In addition, *FBN1* expression levels were correlated with AIS curve severity [18]. Here, patients were divided into two Cobb angle groups (< 45° and ≥ 45°); however, *FBN1* rs12916536 polymorphism was not associated with severity of AIS, only with the development of disease. The total sample size was adequate to detect significant associations with 80% statistical power; the small number of patients in Cobb angle or Lenke classification groups and the biased in term of the age effect because most of AIS cases were skeletally mature with curve Cobb angles exceeding 45° were the main limitations of this study. We believe that the role of *FBN1* polymorphisms in the AIS curve severity and development still needs further investigation. Thus, it is necessary to be accounted for by other variables, such as polymorphisms in *LBX1, GPR126, BNC2, PAX1, LBX1-AS1, BCL2* and *PAX3* genes [6, 8, 10–12, 14], or other yet unknown. Furthermore, no family history data was collected to evaluated the differences between familial and non-familial AIS cases. In addition to providing the first report of the distribution of *FBN1* rs12916536 polymorphism in the heterogeneous Brazilian population and validated the same association observed in Chinese patients, our study has distinct strengths. First, this study reflects real-life community for diagnosing and treating AIS in a public hospital in a developing country. Second, all patients recruited (cases and controls) were evaluated by experienced spine surgeons, excluding spine deformities in the control group.

Whereas AIS is a multifactorial disease, including environmental and genetic factors, it is essential to study this possible association in patients from different populations, including the Brazilian population, which stands out for the extensive admixture and heterogeneity [26, 27]. It is becoming increasingly important to derive data from different populations to validate the role of genetic polymorphisms in AIS and to build a database that can then be used in future investigations (replication study) to better understand and identify modifiable non-modifiable risk factors associated with AIS development and progression.

## Conclusion

In summary, *FBN1* rs12916536 polymorphism play a protective role in the development of AIS, which represents an advance in understanding etiology of disease. Age was an independent risk factor for curve progression. Due to chronic pain, poor health quality of life and the high treatment costs experienced by AIS patient, the genetic information could assist in the diagnosis of the disease, as well as contribute to an individualized treatment for monitoring the at-risk individuals.

## Supplementary Information

**Additional file 1: Supplementary table.** Dataset of case-control study: *FBN1* rs12916536 *G > A* polymorphism associated with susceptibility of adolescent idiopathic scoliosis in Brazilian population. Legend: Age in years. Body mass index (BMI) was calculated as the weight status (kg) divided by the square of height (m^2^). Magnitude of the Cobb angle. The Risser sign was used to estimate skeletal maturity: zero to III were classified as skeletally immature and those with a Risser’s sign of IV or V were classified as skeletally mature.

## Data Availability

The datasets used and/or analysed during the current study are available from the Supplementary material.

## Abbreviations

AIS: Adolescent idiopathic scoliosis
95% CI: 95% Confidence interval
χ2: Chi-square
BMI: Body mass index
FBN1: Fibrillin-1
GWAS: Genome-wide association studies
HWE: Hardy–Weinberg equilibrium
mRNA: Messenger RNA
OR: Odds ratio
PCR: Polymerase chain reaction
R^2^: Degree of correlation
SD: Standard deviation
SPSS: Statistical Package for Social Sciences
